# Preterm infants with isolated cerebellar hemorrhage show bilateral cortical alterations at term equivalent age

**DOI:** 10.1038/s41598-020-62078-9

**Published:** 2020-03-24

**Authors:** Aicha B. C. Dijkshoorn, Elise Turk, Lisa M. Hortensius, Niek E. van der Aa, Freek E. Hoebeek, Floris Groenendaal, Manon J. N. L. Benders, Jeroen Dudink

**Affiliations:** 1 Department of Neonatology, Wilhelmina Children’s Hospital, University Medical Center Utrecht, Utrecht University, Utrecht, the Netherlands; 2 UMC Utrecht Brain Center, Wilhelmina Children’s Hospital, University Medical Center Utrecht, Utrecht University, Utrecht, the Netherlands; 3 Department for Developmental Origins of Disease, Wilhelmina Children’s hospital, University Medical Center Utrecht, Utrecht, the Netherlands

**Keywords:** Paediatric research, Neonatal brain damage

## Abstract

The cerebellum is connected to numerous regions of the contralateral side of the cerebrum. Motor and cognitive deficits following neonatal cerebellar hemorrhages (CbH) in extremely preterm neonates may be related to remote cortical alterations, following disrupted cerebello-cerebral connectivity as was previously shown within six CbH infants. In this retrospective case series study, we used MRI and advanced surface-based analyses to reconstruct gray matter (GM) changes in cortical thickness and cortical surface area in extremely preterm neonates (median age = 26; range: 24.9–26.7 gestational weeks) with large *isolated* unilateral CbH (*N *= 5 patients). Each CbH infant was matched with their own preterm infant cohort (range: 20–36 infants) based on sex and gestational age at birth. On a macro level, our data revealed that the contralateral cerebral hemisphere of CbH neonates did not show less cortical thickness or cortical surface area than their ipsilateral cerebral hemisphere at term. None of the cases differed from their matched cohort groups in average cortical thickness or average cortical surface area in the ipsilateral or contralateral cerebral hemisphere. On a micro (i.e. vertex) level, we established high variability in significant local cortical GM alteration patterns across case-cohort groups, in which the cases showed thicker or bigger volume in some regions, among which the caudal middle frontal gyrus, insula and parahippocampal gyrus, and thinner or less volume in other regions, among which the cuneus, precuneus and supratentorial gyrus. This study highlights that cerebellar injury during postnatal stages may have  widespread bilateral influence on the early maturation of cerebral cortical regions, which implicate complex cerebello-cerebral interactions to be present at term birth.

## Introduction

The cerebellum is fundamental for cognitive and coordinated motor function, varying from sensorimotor mapping to language regulation (as reviewed by Caligiore *et al*.)^1^. Pioneering work in both animal and human studies has established that the cerebellum collaborates with the cerebral cortex and sends output to, and receives input from, contralateral cortical areas via enclosed-loops^2–5^, In human these projection areas include multiple higher-order cerebral regions, such as the dorsolateral prefrontal cortex, the parietal and superior temporal lobes^6–8^. Cerebello-cerebral loops are thought to be anatomically separated and involved in distinct functional processes^1,9^. Many of these efferent and afferent connections have been identified as early as term-equivalent age (TEA) in preterm infants^10–12^.

Cerebellar hemorrhage (CbH) is a common neonatal complication with an ultrasound-detected incidence of 7.6% in extremely preterm infants (gestational age at birth <28 weeks)^13^. In general the most immature and the sickest children are at a higher risk for developing CbH^14,15^, The actual incidence, however, may be higher as it has likely been underdiagnosed clinically. A recent review demonstrates that between 43% and 75% of the preterm (<32 weeks gestation) infants with *isolated* CbH (e.g. absence of supratentorial injury) were severely impaired in cognitive, motor, language and/or behavioral development. On top of extremely preterm birth and other perinatal risk factors, these results suggest that neonatal cerebellar damage make them especially vulnerable for the development of neurodevelopmental defects. This incidence was most seen in infants with vermis involvement (87–93%) and large bleed (46–82%)^16^. Indeed, studies show a strong link between neonatal CbH and autism^14,17–19^. A unified hypothesis that would explain the relation between CbH and neurodevelopmental sequelae in extremely preterm infants is that the cerebellum is part of dynamic brain networks that shape higher order brain function. A loss of innervation from the cerebellum, which could come about due to perinatal complications, may alter the growth of structurally and functionally connected brain regions, such as the thalamus and cortical regions throughout the (pre-) frontal and parietal regions^11,14,20^. In fact, already in the mid-twentieth century it has been observed that cerebral atrophy can follow contralateral cerebellar atrophy and vice versa, especially when the lesions occur in early youth^21^. In accordance with this view Volpe *et al*. (2009) and Wang *et al*. (2014) proposed the term of ‘developmental diaschisis’, as originally used by von Monakow, to explain that damage in one area can ensue damage to remote but connected areas in the context of CbH^14,17,22^. Indeed, one landmark study has found that unilateral injury to the cerebellum in extremely preterm neonates (*N* = 6) can induce volumetric changes in the contralateral uninjured cerebrum at term equivalent age (TEA)^23^.

With advances in neonatal brain MR image processing software it is now possible to accurately reconstruct neonatal cortical surfaces to determine neonatal maturation more precisely. At present we can independently investigate the two components of cortical gray matter volume, i.e. cortical surface area and cortical thickness, which are thought to be driven by distinct multiple genetic mechanisms and could be affected differently by neonatal morbidity^24,25^. To extend our understanding of the impact of CbH on cortical gray matter morphology and its modulation of cerebral functioning we used MRI and surface-based area analysis to identify specific cerebral abnormality patterns in cortical thickness and cortical surface area that could explain CbH-related morbidities. We hypothesized that unilateral CbH in extremely preterm infants would be associated with reduced contralateral cortical thickness and cortical surface area at TEA.

## Materials and methods

### Participants

**Figure 1:**
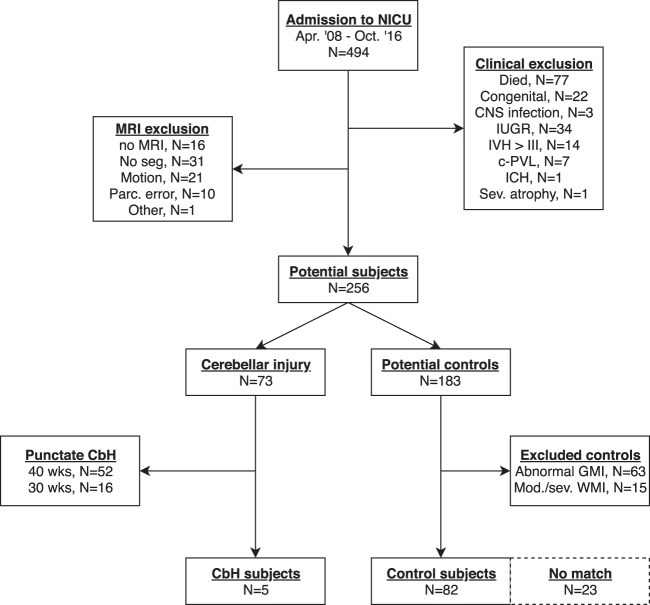
Flowchart of the study design of the matched case-cohort groups. NICU = neonatal intensive care unit, Congenital = congenital anomalies, CNS = central nervous system, IUGR = intrauterine growth restriction (<10^th^ percentile), IVH = intraventricular hemorrhage, c-PVL = cystic periventricular leukomalacia, ICH = intracranial hemorrhage, MRI = magnetic resonance imaging, Seg = segmentation, Parc = parcellation, CbH = cerebellar hemorrhage, Wks = weeks, GMI = gray matter injury, WMI = white matter injury, Mod = moderate, Sev = severe. Intraventricular hemorrhages were score according to Papile *et al*. (1978). White and gray matter injury was assessed according to Woodward *et al*. (2006). Cerebellar hemorrhages were scored according to Kidokoro *et al*. (2014).

A total of 494 preterm infants born <28 weeks of gestational age admitted to the Neonatal Intensive Care Unit (NICU) of the Wilhelmina Children’s Hospital (Utrecht, Netherlands) between April 2008 and October 2017 were eligible for participation in this retrospective study. We included infants with large (defined as bleeds >3 mm) isolated CbH (e.g. absence of associated supratentorial lesions) and a cohort of matched control preterm born infants that underwent a successful MRI scan at TEA (40 postmenstrual weeks)^26^. Exclusion criteria were death, congenital anomalies, central nervous system diseases, intrauterine growth restriction (<10^th^ percentile), intraventricular hemorrhages > grade III as identified by Papile *et al*. (1987), intracerebral hemorrhages and cystic periventricular leukomalacia^27^. We also excluded potential control infants with abnormal gray matter injury (a score of 6 or higher) and moderate or severe white matter injury (a score of 10 or higher) using the Woodward score^28^. Brain injury evaluations were done by a pediatric neuro-radiologist and an experienced neonatologist (MB) with more than ten years of experience in neonatal neuro-imaging. We excluded potential CbH infants with punctate CbH (defined as bleeds <3 mm) on MR images at 30 weeks and at 40 weeks according to the cerebellar injury score by Kidokoro *et al*.^26^. Based on these criteria five infants with large isolated CbH and 82 control infants were eligible for inclusion of this study. The use of clinically obtained data for scientific inquiries was approved by the Institutional Review Board (IRB) of the University Medical Center Utrecht, the Netherlands. Because we only used clinically acquired data, informed consent by the parents for study participations was waived by the IRB. The data extraction procedures have been performed in accordance with the ethical standards as laid down in the 1964 Declaration of Helsinki and its later amendments or comparable ethical standards. Clinical characteristics of the selected study populations are outlined in Table 1 and the flow diagram for exclusion is supplied in Fig. 1.

**Table 1:** Clinical variables of the five CbH cases and their matched cohort groups.

	CbH#1	CG#1	CbH#2	CG#2	CbH#3	CG#3	CbH#4	CG#4	CbH#5	CG#5
**Clinical variables**										
Controls, no	—	20	—	29	—	36	—	29	—	26
Sex	Female	Female	Male	Male	Female	Female	Male	Male	Male	Male
Gestational age, in weeks	24.9	25.2 (0.50)	26	26 (0.58)	26.7	26.7 (0.62)	26.3	26.2 (0.52)	25.6	25.8 (0.55)
Birth weight, in percentiles	71th	68th (22)	43th	63th (20)	98th	64th (19)	94th	63th (20)	63th	64th (19)
PMA at scan, in weeks	41.2	41.1 (0.77)	40.9	41.3 (0.76)	41.2	41.1 (0.60)	42.1	41.2 (0.75)	40.4	41.3 (0.69)
Days of ventilation	NA	10.8 (13)	22*	6.8 (5.7)	17	6.3 (5.8)	14	8.6 (7.9)	12	9 (5.7)
Apgar score at 5 min	7	7.5 (1.0)	6	6.9 (1.5)	8	7.3 (1.6)	9	6.9 (1.6)	6	6.8 (1.6)
**Brain injury**										
Type CbH	UN + vermis right	—	UN + vermis left	—	UN left	—	UN left	—	UN right	—
IVH, no (%)										
None	—	—	1 (100)	19 (64)	—	28 (73)	—	21 (69)	—	17 (66)
Grade I	1 (100)	20 (100)	—	4 (14)	—	3 (10)	—	3 (12)	—	4 (15)
Grade II	—	—	—	5 (18)	1 (100)	3 (10)	—	4 (15)	1 (100)	5 (19)
Grade III	—	—	—	1 (4)	—	2 (7)	1 (100)*	1 (4)	—	—
Total WMI score	9	8.1 (0.85)	10*	7.4 (1.2)	8	7.5 (1.3)	8	7.3 (1.2)	10*	7.5 (1.2)
Total GMI score	6*	4.4 (0.69)	4	4.5 (0.64)	5	4.5 (0.56)	5	4.4 (0.70)	3*	4.5 (0.59)

Continuous data are depicted in mean (standard deviation) in case data are normally distributed or median (range) otherwise. Binary clinical outcome measures are depicted as number (%). Independent Sample Students t-test were performed between each CbH infant and their matched cohort group for continuous normally distributed data. The nonparametric Mann-Whitney U test was used for binary clinical outcome data. CbH = cerebellar hemorrhage, CG = control group, GMI = gray matter injury, IVH = intraventricular hemorrhage, NA = not assessed, No = number, PMA = postmenstrual age, UN = unilateral, WMI = white matter injury. Intraventricular hemorrhage was scored according to Papile *et al*. (1987). White and gray matter injury was assessed according to Woodward *et al*. (2006). *p < 0.05.

### Study design and rationale

In order to answer our research question, we applied three strict exclusion criteria. First of all we excluded all infants with severe prematurity-related complications that are known to affect neurodevelopment and mortality; congenital malformations, central nervous system infections, intrautering growth restriction, intraventricular hemorrhage > III, cystic periventricular leukomalacia and intracerebral hemorrhage^29–31^. We also excluded one infant in which the radiologist noted severe atrophy. These complications were extracted from the numerous studies which have compared cortical development in preterm infants compared to term born infants^32,33^. Accordingly, this study will focus on the impact of a CbH on cortical development on top of the vulnerability of being extremely premature. Secondly, we excluded control infants with supratentorial injuries (e.g. white matter injury and gray matter injury). To our knowledge, only one study has yet found that unilateral (e.g. one side of the cerebellum) CbH results in reduced contralateral (e.g. opposite side of the cerebellum) cerebral cortical volume. No effects were found in the cerebral cortex ipsilateral to the side of the cerebellum damage^34^. Thirdly, as this represents a remote effect in an already vulnerable brain, we excluded all infants with punctate CbH. Application of these exclusion criteria resulted into the inclusion of 83 control infants and five CbH infants.

Given the knowledge that the severity of prematurity (e.g. born at 24 weeks versus 27 weeks) is related to cortical development and given the theorem that different areas of the cerebellum project to distinct cortical areas, we did not group the CbH infants together. Instead, we matched each infant with a CbH to a maximum number of control infants with the same sex and gestational age at birth (+/−1 week). We then additionally controlled for birth weight z-score, postmenstrual age at scan and gestational age at birth. Though some studies have indicated 10–15 cases per parameter to reliably estimate regression coefficients, we believed that because of the extreme vulnerability of this population the likelihood that these covariates would better explain possible alterations in cortical development would be too high^14,35^. We therefore chose a more conservative approach in favor of a decreased likelihood for third explanatory variables. Each CbH infant ultimately matched with 20 to 36 control infants (Table 1). In the following sections the case-cohort groups will be represented as following; an infant with CbH in comparison to that infants’ matched control cohort will be CbH#1 and CG#1, respectively, thereby referring to the first CbH infant as presented in Table 1. If we are referring to the second CbH infant and its control cohort, we will use CbH#2 and CG#2, and so forth.

### MRI data acquisition

According to clinical protocol the extremely preterm infants were scanned at 30 weeks of gestation if clinically stable, and around TEA. All scans were acquired on a 3.0 Tesla MR system (Philips Healthcare, Best, Netherlands) using an 8-channel sense head coil. Prior to scanning, infants at TEA were sedated with 50–60 mg/kg oral chloral hydrate according to clinical protocol and wrapped into a vacuum cushion pillow to minimize motion (Kohlbrat an Bunz GmbH, Radstadt, Austria). Minimuffs were used for hearing protection (Natus Medical Inc. San Carlos, CA, USA; Em’s Kids LLC, Culver City, CA, USA). For our analysis we used the scan at TEA. The scan protocol included the acquisition of 3D coronal T2-weighted images (T2-weighted repetition time 4847 ms; echo time 150 ms; total scan time 5.05 min; in plane FOV 180 × 180 mm; acquisition matrix 232 × 202 mm; number of sections 110; in-plane spatial resolution 0.35 × 0.35 mm; voxel size 0.78 × 0.89 with 1.2 mm slice thickness; no gap).

### MR image processing

**Figure 2:**
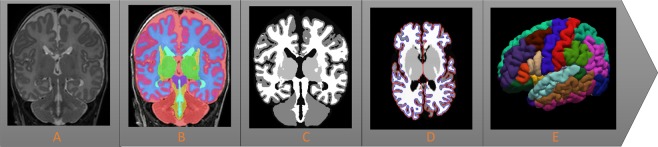
Simplified overview of the neonatal pipeline for magnetic resonance imaging processing. (**A**) A neonatal coronal T2-weigthed image at term equivalent age. (**B**) The same T2-weighted image is segmented into eight tissue types. (**C**) The segmented brain tissue image serves as a template to artificially reconstruct a T1-weighted image, by assigning tissue intensity values that can be read by Freesurfer’s adult-driven pipeline. (**D**) The resulting surface-based extraction by Freesurfer, in which the blue line represents the inner surface between the white and gray matter and the yellow line represents the outer surface between the gray and pial matter. (**E**) The reconstruction of the cortical mantle and its parcellation into different regions that corresponds to the Desikan-Killiany atlas.

Freesurfer version 5.3 Suite software (https://surfer.nmr.mgh.harvard.edu/) was used to automatically process and reconstruct the cortical surface of T1-weighted images^36^. Before reconstruction of the cortical mantle we (1) used the automated segmentation method as described by Moeskops *et al*. (2016) that automatically segmented T2-weighted images (Fig. 2a) into eight tissue classes (Fig. 2b)^37^. This method has been evaluated and scored best by the Neonatal Brain Segmentation challenge (NeobrainS12)^38^. (2) The resulting segmentations were visually inspected and manually edited in case of minor voxel misclassifications (<10% missegmentations). (3) We reconstructed artificial T1-weighted images by assigning inverted tissue intensity values at each tissue class on the T2-weighted neonatal segmentations images (Fig. 2c). (4) This image was given as input into the Freesurfer pipeline (Fig. 2d,e).

The technical details of the procedures through which Freesurfer creates a three-dimensional cortical surface has been described in great detail by prior publications^39,40^. According to Freesurfers’ protocol, the white and pial surface extractions (Fig. 2d) were visually inspected. Ten images were discarded due to parcellation errors (Fig. 1). From the reconstructed cortical mantle, we extracted cortical thickness and pial surface area. Cortical thickness is a brain measure used to describe the combined thickness of the layers of the cerebral cortex. It was operationalized as average shortest distance between the white and pial matter surface on a vertex-by-vertex basis. The cortical surface area is a brain measurement that states the total area that is covered by the outer layer of the cerebral cortex, which was defined as geometric center between the inner and outer surfaces. The cortical thickness and cortical surface area was analyzed on a micro level (vertex-by-vertex basis) and on a macro level (average cortical thickness and cortical surface area per hemisphere). Based on gyral and sulcian landmarks the cortical mantle could then be automatically parcellated into 34 adult-based anatomical regions for each hemisphere^41^. These adult-based atlases are based on cortical folding information, of which the rough outline is already present in extremely preterm infants at TEA^42^. With the additional described manual steps (step 2 and 3) we could visually verify that Freesurfer was able to apply the intensity-based tissue segmentation masks to create the fitting cortical surface-based extractions and the reconstructed cortical mantle. To reduce noise-induced variations a full width half maximum Gaussian blurring kernel of 15 mm was applied to smooth the surfaces, comparable to previous research in preterm children^43,44^.

### Statistical analysis

We conducted paired sample Student’s *t*-tests to investigate whether CbH had an effect on asymmetric (contralateral vs. ipsilateral) development of the cortex within the CbH patient group, using IBM SPSS statistics version 22 (IBM, Armonk, New York). To investigate asymmetric development per region of interest Analysis of Covariance (ANCOVA) was performed. Diverse cortical alteration patterns were found across regions of interest (data not shown, available upon request). Independent sample Student’s *t*-tests were used to examine clinical group differences for continuous normally distributed data, that is for birth weight, post menstrual age at scan, days of ventilation, Apgar score at 5 minutes, total white matter injury score and total gray matter injury score. We applied the nonparametric Mann-Whitney *U* test regarding the presence or absence of intraventricular hemorrhage (binary outcome data).

We performed the independent sample Student’s *t-*test for matched case-cohort groups analysis in average cortical thickness and average cortical surface area per hemisphere (contralateral versus ipsilateral). To account for multiple testing of the latter, we considered a *p*-value of <0.005 (0.05/10 tests) significant (5 case-cohort combinations × 2 cortical outcome measures). The General Linear Model approach was executed using Freesurfers’ QDEC software, to perform a vertex-wise analysis across the entire brain to investigate differences in cortical thickness and cortical surface area between the matched case-cohort groups. The linear model was created with a Different Onset Same Slope method and postmenstrual age at scan, gestational age at birth and birth weight z-score were brought into the model as demeaned covariates. We then applied a false discovery rate (FDR) to correct for multiple comparisons. The resulting difference maps show statistically significant differences if they survived FDR correction of 0.05. Because the FDR-correction might be considered too conservative, we conducted a hypothesis generating analysis for which we considered a *p*-value of <0.001 significant. Clusters of significant vertex-base changes were labelled with reference to the Desikan-Killiany atlas.

## Results

### Sample characteristics

**Figure 3:**
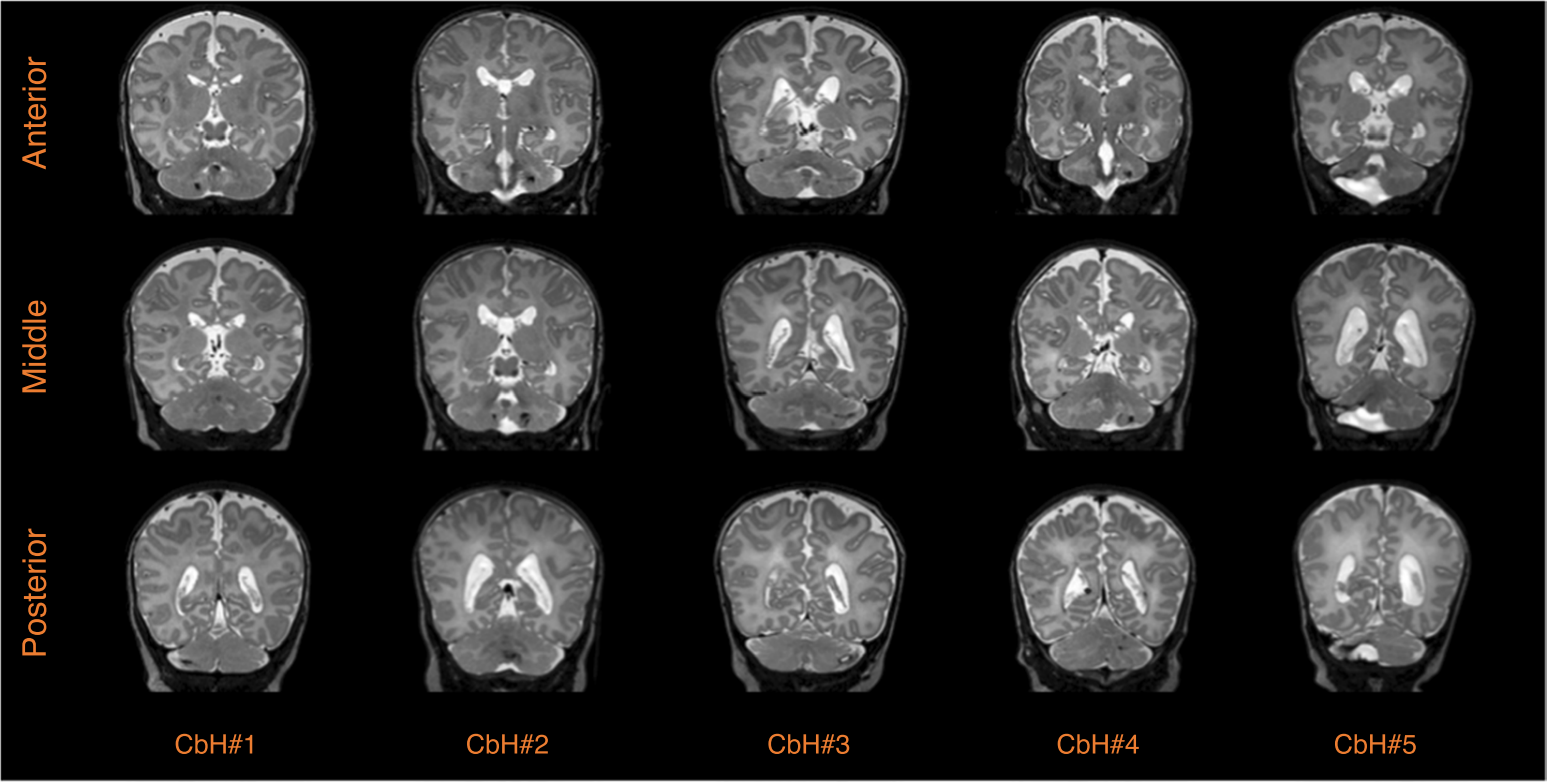
Coronal T2-weighted images on the size and location of the hemorrhages in the five infants with CbH at term equivalent age. To capture the three-dimensionality of the hemorrhages we provided a frame of the anterior, the middle and the posterior part of the hemorrhage.

Five preterm CbH infants and 82 preterm control infants were eligible for inclusion in the present study. The location of the CbH differed between the five subjects (Fig. 3). Three patients had large unilateral bleeds in a hemisphere (two left, one right) and two patients had large unilateral bleeds that included the vermis (one left, one right) (Fig. 3). As pointed out in Table 1, the matched case-cohort groups were comparable on clinical variables, except for the CbH infant in group two, who needed significantly longer ventilation, *p* = 0.015. An independent sample Student’s *t*-test further revealed differences on brain injury scores between the patients and their matched cohort groups. Importantly, from the five infants with a large CbH, the CbH infants in case-cohort 1, 2 and 5 had scored significantly higher on gray matter injury or white matter injury scales and for the CbH infant in case-cohort 4 a significantly higher intraventricular hemorrhage grade was reported than their matched cohort groups. Thus, the infants with CbH more often showed patterns of brain injury. Despite not being significantly different from the control cohort, in the context of this study it may also be relevant to note that the CbH infant in group 2 and 3 are relatively large for their GA, with a BW-z in the 98^th^ percentile and the 94^th^ percentile, relatively. In addition, the Apgar score at 5 min was low (less than 7) for the CbH infant in group 2 and 5.

### Whole brain analysis

We first assessed whether there were lateralization effects of total cortical volume per hemisphere within the five CbH subjects. The within-subject differences of the cases are presented in Table 2. The paired sample Student’s *t*-test revealed that the contralateral cortex was not significantly thinner, *p* = 0.32, nor had a smaller surface area, *p* = 0.61, than the ipsilateral hemisphere within the CbH group. Next, we obtained the mean cortical thickness and the mean cortical surface area of the hemispheres for the different matched case-cohort groups as provided in Table 2. Although we established variability between the matched case and cohort groups, the independent Student’s *t*-test demonstrated no significant (*p* < 5_E_^−3^ (significance level of 0.05/10 tests to account for multiple testing)) group differences in the average cortical thickness or in the average cortical surface area in neither the ipsilateral nor the contralateral hemisphere.

**Table 2:** Whole brain analysis: average cortical thickness and pial surface area of the CbH infants and their matched cohort groups.

		CbH#1	CG#1	*Sig*.	CbH#2	CG#2	*Sig*.	CbH#3	CG#3	*Sig*.	CbH#4	CG#4	*Sig*.	CbH#5	CG#5	*Sig*.
Thick- ness (in mm)	Ipsi-lateral	1.48	1.46 (0.004)	0.42	1.46	1.47 (0.003)	0.65	1.49	1.47 (0.005)	0.37	1.50	1.46 (0.003)	0.03	1.47	1.46 (0.003)	0.58
	Contra-lateral	1.46	1.46 (0.004)	0.92	1.45	1.46 (0.004)	0.34	1.50	1.46 (0.004)	0.20	1.49	1.46 (0.004)	0.14	1.48	1.47 (0.004)	0.45
Surface area (in cm^2^)	Ipsi-lateral	403	422 (6.5)	0.52	432	449 (8.7)	0.74	444	435 (5.5)	0.78	435	454 (10)	0.73	376	450 (9.3)	0.14
	Contra-lateral	396	422 (6.4)	0.39	448	448 (8.9)	0.75	435	535 (5.7)	0.99	442	454 (10)	0.83	377	450 (9.1)	0.14

Values are displayed as Mean (Standard Error). CbH = cerebellar hemorrhage infant, CG = control cohort, sig = significance.

*p < 0.005.

### Vertex base analysis

**Figure 4:**
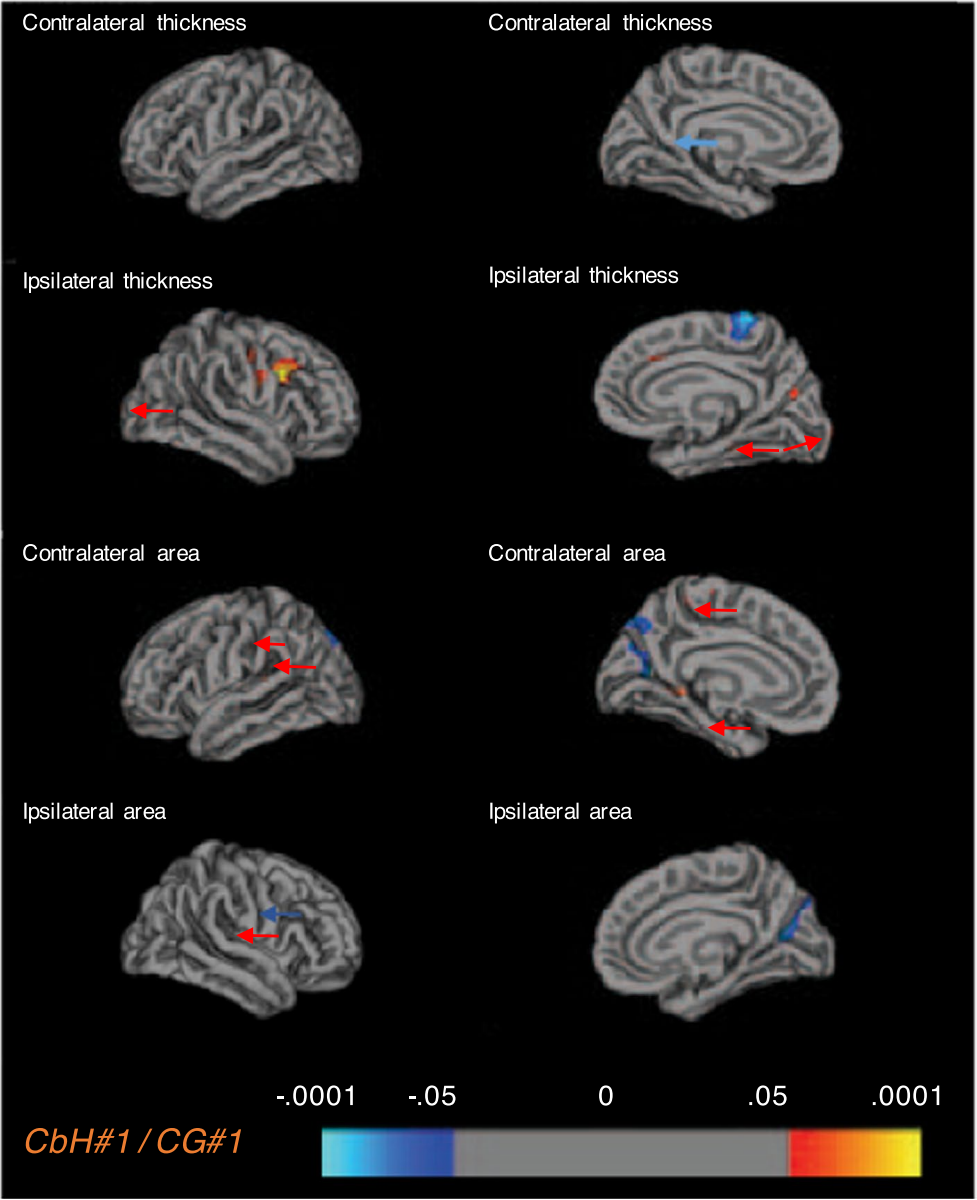
Alterations in cortical thickness and pial surface area in the matched case-cohort groups. Significantly different areas are shown in color, blue to light blue represents a thinner (less area) cortex in the CbH infants, whereas red to yellow represents a thicker (more area) cortex. The color scale reflects the strength of the *p*-value (FDR-corrected). Colored arrows are included for visually hidden areas. Dark gray = sulci, Light gray = gyri.

**Figure 5:**
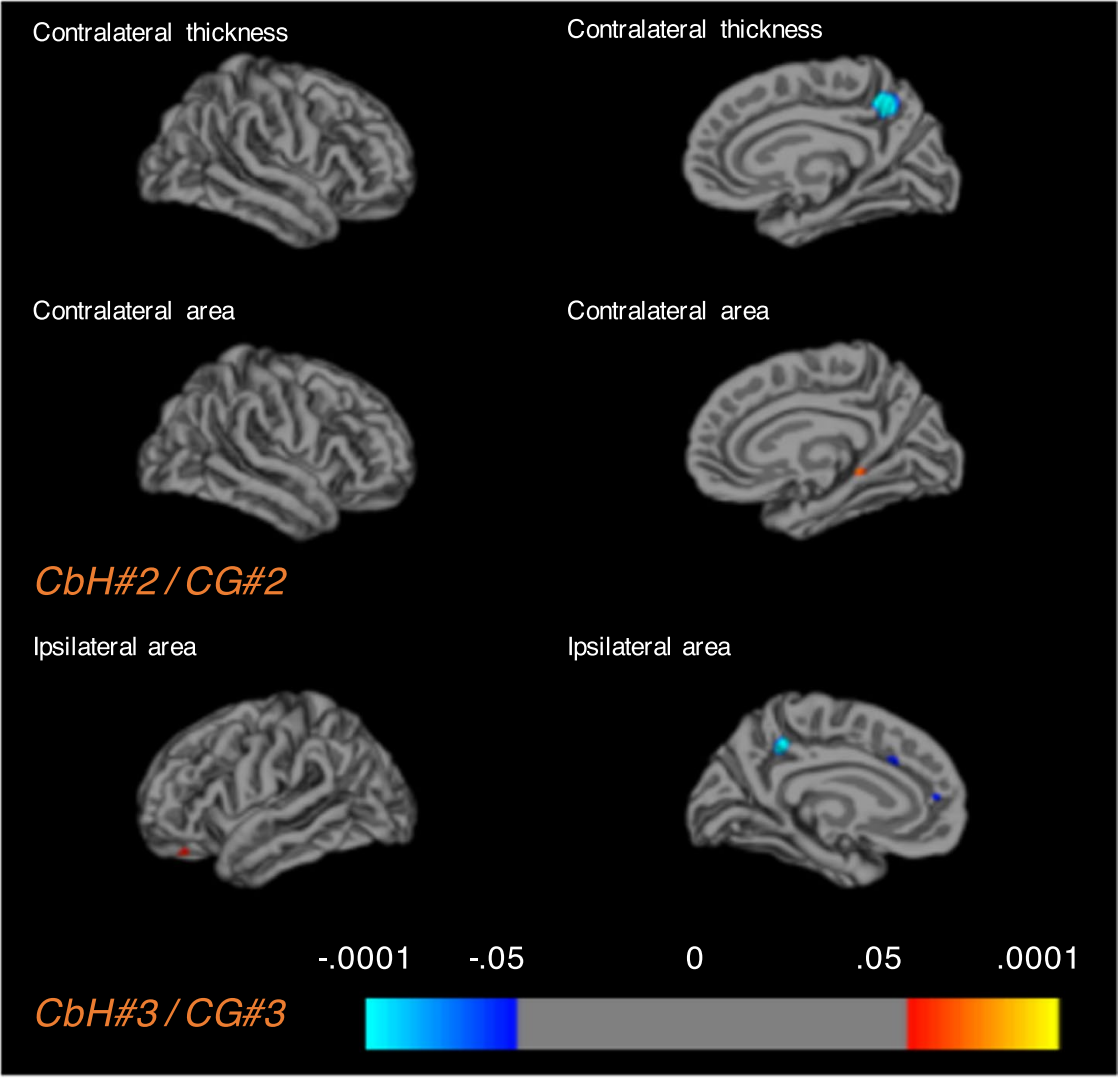
Alterations in cortical thickness and pial surface area in the matched case-cohort groups. Significantly different areas are shown in color, blue to light blue represents a thinner (less area) cortex in the CbH infants, whereas red to yellow represents a thicker (more area) cortex. The color scale reflects the strength of the *p*-value (FDR-corrected). Colored arrows are included for visually hidden areas. Dark gray = sulci, Light gray = gyri.

We then assessed whether there were differences in cortical thickness and cortical surface area between the matched case and cohort groups on a vertex-base level. We found diverse cortical alteration patterns throughout the brain, which varied across case-cohort groups (Table 3). Of the five CbH cases, three infants showed FDR-corrected significant areas (thicker or bigger volume (+), thinner or less volume(−)) when compared to their unique cohort groups. In the first CbH case the following areas differed in the contralateral hemisphere; insula (+), ishmuscingulate gyrus (+), the ishmuscingulate gyrus (−), paracentral lobule (+), postcentral gyrus (+), and in the ipsilateral hemisphere; caudal middle frontal gyrus (+), cuneus (−), fusiform gyrus (+), insula (+), middle frontal gyrus (−), postcentral gyrus (+), precuneus (+), superior frontal gyrus (+), superior temporal gyrus (−). In the second CbH infant the contralateral parahippocampal gyrus (+) and the precuneus (−) differed and in the third CbH infant the ipsilateral precuneus (−) and superior frontal gyrus (−). Only comparison of case-cohort groups that resulted in significant FDR-corrected areas are presented in Fig. 4 and in Fig. 5. Most significantly, the *contralateral* precuneus was respectively thinner (*p* = 5_E_^−11^) in the infant with the unilateral left CbH with vermis involvement (Fig. 5, CbH#2 vs. CG#2) and the *ipsilateral* precuneus was respectively thinner (*p* = 9_E_^−13^) in the infant with the unilateral left CbH (Fig. 5, CbH#3 vs. CG#3). There were no other FDR-corrected areas across the case-cohort groups. The less conservative, hypothesis generating analysis (using *p* < 0.001) showed diverse cortical alteration patterns in all five case-cohort groups. An overview of the significant clusters is added in Table 3. Please note that cortical thickness and surface volumes provided are based on the whole cortical region and that thinning or thickening values are based on specific differences set of clusters within a ROI. These numbers indicate that in 88% of the case-cohort comparisons the direction of the effect is similar. We found more often differences in cortical thickness than in the cortical surface area. In addition, more cortical areas were significantly thicker, rather than thinner.

**Table 3:** Whole brain vertex-based analysis in the matched case-cohort groups with significant clusters set at *p* < 0.001.

	CbH#1/CG#1				CbH#2/CG#2				CbH#3/CG#3			
	Cortical Cluster	CbH#1	CG#1	*Sig*.	Cortical Cluster	CbH#2	CG#2	*Sig*.	Cortical Cluster	CbH#3	CG#3	*Sig*.
***Contralateral***												
Thinning (in mm)	ICgC*	0.96	1.39 (0.08)	3_E_^−06^	PCUN*	0.81	1.45 (0.07)	5_E_^−11^	—	—	—	—
Less surface area (in mm^2^)	CUN*	0.22	0.44 (0.03)	1_E_^−06^	CUN	0.26	0.61 (0.08)	7_E_^−05^	—	—	—	—
	SPG*	0.19	0.32 (0.02)	9_E_^−07^	—	—	—	—	—	—	—	—
Thickening (in mm)	cMFG	1.60	1.44 (0.04)	6_E_^−04^	—	—	—	—	FFG	1.64	1.39 (0.07)	1_E_^−04^
	PoCG	1.56	1.40 (0.04)	1_E_^−04^	—	—	—	—	LING	1.50	1.28 (0.05)	7_E_^−04^
	—	—	—	—	—	—	—	—	PreCG	1.58	1.34 (0.04)	3_E_^−04^
	—	—	—	—	—	—	—	—	—	—	—	—
More surface area (in mm^2^)	INS*	0.26	0.15 (0.02)	6_E_^−05^	PHG*	0.48	0.21 (0.06)	2_E_^−06^	—	—	—	—
	ICgC*	0.30	0.16 (0.02)	4_E_^−05^	STG	0.34	0.25 (0.03)	4_E_^−04^	—	—	—	—
	PCL*	0.36	0.24 (0.03)	1_E_^−04^	—	—	—	—	—	—	—	—
	PHG	0.33	0.22 (0.03)	5_E_^−04^	—	—	—	—	—	—	—	—
	PoCG*	0.28	0.21 (0.01)	2_E_^−04^	—	—	—	—	—	—	—	—
***Ipsilateral***												
Thinning (in mm)	PCL	1.36	1.63 (0.04)	9_E_^−06^	—	—	—	—	PCUN*	0.88	1.38 (0.05)	9_E_^−13^
	—	—	—	—	—	—	—	—	SFG*	0.61	1.44 (0.23)	1_E_^−04^
	—	—	—	—	—	—	—	—	SFG	1.04	1.51 (0.13)	4_E_^−04^
Less surface area (in mm^2^)	CUN*	0.26	0.53 (0.04)	7_E_^−07^	—	—	—	—	—	—	—	—
	STG*	0.17	0.24 (0.02)	3_E_^−04^	—	—	—	—	—	—	—	—
Thickening (in mm)	cMFG*	1.81	1.37 (0.05)	2_E_^−06^	—	—	—	—	LOC	1.87	1.55 (0.07)	1_E_^−04^
	FFG*	1.71	1.34 (0.07)	2_E_^−04^	—	—	—	—	LING	1.88	1.56 (0.08)	3_E_^−04^
	INS*	1.59	1.32 (0.04)	1_E_^−08^	—	—	—	—	PreCG	1.68	1.40 (0.07)	6_E_^−04^
	LOCC*	1.77	1.55 (0.05)	2_E_^−04^	—	—	—	—	—	—	—	—
	LING*	1.54	1.36 (0.04)	1_E_^−04^	—	—	—	—	—	—	—	—
	IFGtriang	1.63	1.32 (0.06)	3_E_^−04^	—	—	—	—	—	—	—	—
	PoCG*	1.73	1.42 (0.06)	7_E_^−04^	—	—	—	—	—	—	—	—
	PreCG	1.66	1.34 (0.08)	9_E_^−05^	—	—	—	—	—	—	—	—
	PCUN*	1.69	1.38 (0.06)	9_E_^−05^	—	—	—	—	—	—	—	—
	SFG*	1.81	1.47 (0.07)	5_E_^−04^	—	—	—	—	—	—	—	—
More surface area (in mm^2^)	cMFG*	0.34	0.17 (0.03)	2_E_^−05^	—	—	—	—	—	—	—	—
	**CbH#4/CG#4**				**CbH#5/CG#5**							
	**Cortical Cluster**	**CbH#4**	**CG#4**	***Sig****.*	**Cortical Cluster**	**CbH#5**	**CG#5**	***Sig****.*				
***Contralateral***												
Thinning (in mm)	PCUN	0.75	1.32 (0.12)	3_E_^−05^	ICgC	1.07	1.58 (0.11)	7_E_^−04^				
Less surface area (in mm^2^)	PreCG	0.15	0.27 (0.04)	7_E_^−04^	—	—	—	—				
	—	—	—	—	—	—	—	—				
Thickening (in mm)	IFGoperc	1.73	1.52 (0.06)	4_E_^−04^	CUN	1.73	1.50 (0.06)	4_E_^−05^				
	—	—	—	—	LOCC	1.80	1.50 (0.06)	1_E_^−04^				
	—	—	—	—	PCUN	1.77	1.49 (0.05)	1_E_^−04^				
	—	—	—	—	SMG	1.77	1.49 (0.05)	2_E_^−04^				
More surface area (in mm^2^)	—	—	—	—	—	—	—	—				
	—	—	—	—	—	—	—	—				
	—	—	—	—	—	—	—	—				
	—	—	—	—	—	—	—	—				
	—	—	—	—	—	—	—	—				
***Ipsilateral***												
Thinning (in mm)	PoCG	1.32	1.46 (0.07)	3_E_^−04^	—	—	—	—				
	—	—	—	—	—	—	—	—				
	—	—	—	—	—	—	—	—				
Less surface area (in mm^2^)	—	—	—	—	—	—	—	—				
	—	—	—	—	—	—	—	—				
Thickening (in mm)	FFG	1.72	1.46 (0.08)	5_E_^−04^	ORBmid	1.69	1.50 (0.05)	3_E_^−04^				
	IPG	1.68	1.44 (0.06)	2_E_^−05^	SFG	1.72	1.53 (0.05)	1_E_^−04^				
	LOCC	1.95	1.54 (0.07)	4_E_^−05^	SPG	1.64	1.41 (0.04)	8_E_^−05^				
	PoCG	1.57	1.41 (0.05)	2_E_^−04^	—	—	—	—				
	—	—	—	—	—	—	—	—				
	—	—	—	—	—	—	—	—				
	—	—	—	—	—	—	—	—				
	—	—	—	—	—	—	—	—				
	—	—	—	—	—	—	—	—				
	—	—	—	—	—	—	—	—				
More surface area (in mm^2^)	—	—	—	—	—	—	—	—				

cMFG = caudal middle frontal gyrus, CUN = cuneus, FFG = fusiform gyrus, ICgC = Ishmuscingulate gyrus, IFGoperc = inferior frontal gyrus – opercular part, IFGtriang = inferior frontal gyrus – parstriangular part, INS = insula, IPG = inferior parietal gyrus, LING = lingual gyrus, LOC = lateral orbitofrontal cortex, LOCC = lateral occipital cortex, ORBmid = Middle frontal gyrus - orbital part, PCL = paracentral lobule, PCUN = precuneus, PHG = parahippocampal gyrus, PoCG = postcentral gyrus, PreCG = precentral gyrus, SFG = superior frontal gyrus, SMG = supramarginal gyrus, STG = superior temporal gyrus, STGbank = banks of superior temporal gyrus, SPG = superior parietal gyrus.

*Significant after FDR (0.05) correction.

## Discussion

This is, to the best of our knowledge, the first study to separately investigate cortical thickness and cortical surface area in extremely preterm neonates with an isolated CbH. In contrast to our hypothesis, our data reveal that CbH is not inevitably related to contralateral cortical thinning or surface area loss at TEA neither within the CbH infant group nor when compared to their seperately matched control cohorts. Instead we find bilateral cortical alteration patterns of increased and decreased cortical thickness and cortical surface area that varied across matched case-cohort groups. This study highlights that the developing cerebello-cerebral interactions may involve many regions outside of the sensorimotor cortices in both hemispheres, underlining the complexity of the development of the interconnected neural networks. Most likely the cerebello-cerebral relationship is a mutifaceted interplay between spatial and temporal aspects, such as the size and location of the hemorrhage, but also the clinical mediators and confounders, such as the degree of immaturity, mild supratentorial brain injury or perinatal stressful events^45^.

Our results did not indicate uniform contralateral cortical thinning or surface area loss after unilateral CbH (with vermis involvement) at TEA. Because this is the first study to extract cortical thickness and cortical surface area in extremely preterm neonates this finding is discussed in light of studies assessing diverse aspects or time points of brain and cerebellar growth. Using voxel-based morphometry (VBM), Limperopoulos *et al*. (2005) found contralateral cerebral brain volume loss in preterm infants (Mean GA = 26.7 weeks, SD = 2.3) with large CbH (Four right CbH vs. two left CbH) at term^23^. Visual comparison between our studies suggest that the CbH in our cohort were relatively smaller, which could be an influential factor in the observed cortical morphology. In adult patients with cerebellar injury, using VBM, Clausi *et al*. (2007) showed diminished cortical gray matter volume occurred only after right cerebellar injury^46^. Conversely, another study in adult patients with cerebellar injury showed bilateral decreases in cortical gray matter volume^47^. Taken together, it remains to be elucidated whether there might be a link between location and size of the CbH and specific cortical alteration patterns within our cohorts.

Furthermore, in agreement with microstructural diffusion tensor imaging research that demonstrated the association between isolated preterm CbH and widespread bilateral cerebello-cerebral circuit alterations^48^, we showed that isolated preterm CbH is linked to widespread cortical gray matter alteration patterns that include bidirectional changes in cortical thickness and cortical surface area. There are several, not mutually exclusive, explanations for the exposed patterns^49,50^. The location of the CbH may influence the variability of the patterns, because they can implicate different cerebello-thalamo-cerebral loops. We found the most significant case-cohort differences in the CbH infants with vermis involvement. Interestingly, a recent systematic review revealed that damage in the vermis is associated with severe neurodevelopmental disability in multiple outcome domains^16^. Vermal cerebellar cortex projects to the fastigial nucleus from which axons cross at the decussation and project to the contralateral intralaminar, ventromedial and mediolateral geniculate nuclei of the thalamus and subsequently diffusely project to layers of the contralateral cerebral cortex^51,52^. In contrast to the globosus, emboliformus and dentatus nuclei, estimated three-quarters of the axons within the fastigial nucleus also cross intracerebellar to form the hook bundle^53,54^. This could subsequently explain why infants with CbH with vermis involvement reveal a greater variety in diffuse and bilateral cerebral cortical alteration patterns. In addition, it is probable that other confounding and mediating factors are implicated in the cerebello-cerebral developmental relationship and cortical surface-based morphology. For instance, we found the most pronounced cortical surface differences in the precuneus in two matched case-cohort groups (Fig. 5). The precuneus is mainly involved in episodic memory retrieval and mental imagery, self-processing and conscious representation of information in forms of spontaneous thoughts and mental images, but also its manipulation for planning or problem-solving purposes^55^. Moreover, it forms a network with higher-association cortical structures, such as the prefrontal, superior temporal and parietal areas^55,56^. As such, this is an important area for highly integrated and associative information processing^55,57^. However, since this region was both ipsi- and contralaterally affected after a CbH, and the anatomical connections of the cerebellar hemispheres are believed to be predominantly contralateral, more clinical variables (e.g. amount of supratentorial brain injury, perinatal stressful life events) are thought to be implicated^8^.

It may be that well-defined and uniform cortical thickness and cortical surface area patterns are not yet detectable on a macroscopical level at TEA, because cerebellar growth and cerebellar neuronal differentiation continues to develop well into the second postnatal year^58,59^. Concomitantly, the development of the cortical gray matter also remains heterogeneous and regionally dynamic^60–62^. Therefore, cortical thickness and cortical surface area alterations might become more evident later in development. Longitudinal research on the development of the cortical gray matter will further elaborate our understanding on whether the diverse cortical difference patterns will become more distinct with increasing age, or whether group differences in cortical morphology are inevitable and can be understood best from a network-based approach.

The strengths of this study include the strict in- and exclusion criteria for study eligibility, careful applied matching criteria for the case-cohort groups, application of innovative MR and surface-based analysis used on individual patients rather than group average data and the use of a largely automated pipeline therefore promoting observer independence and reproducible data-processing. When interpreting the results of our study several limitations need to be taken into account. First, we could include only five patients with a *large* isolated CbH that represents a very small sample size. However, we have chosen to apply strict exclusion criteria to ensure a fairly homogenous group. Within these cases we carefully applied matching to promote valid comparison. Nonetheless, we might not have had the statistical power to detect clear cortical alteration patterns. A larger sample would further allow the power to detect the full impact of a wider range of potential confounders and mediators and could potentially reveal subgroup differences. Second, Freesurfer is originally developed for children and adults and has not yet been validated for neonatal images. Despite the quality control steps and the visual verification of an accurate reconstructed cortical mantle, neonatal age-specific atlases would improve parcellation accuracy as the cortex continuous to develop postnatally as well^63^. Third, we only reviewed the gray matter cortical surface on a macroscopical level at TEA. To understand the interplay between timing and circumstances on the cerebello-cerebral developmental processes we would need research including different modalities (e.g. microstructural, functional and connectome-based) as well as longitudinal studies. In addition, corroborating our findings in healthy term-born infants would be valuable in order to elaborate our understanding on the cerebello-cerebral relationship in a typically developing brain. This would broaden our perspective, as it is possible that preterm birth in and of itself could disrupt the typical developmental time course during the third trimester even in absence of notable lesions on MR.

The interpretation of the differences between case-cohort groups is not straightforward. For instance, homologous regions of the ipsilateral hemisphere might be included in a broader neuronal network that indirectly, for instance via the corpus callosum, suffers from the functional disconnection of the cerebellum^46^. This hypothesis lends support from studies that suggest that the injury is not confined to the site where it initially occurred and that it may result in experience-dependent structural remodeling of the entire cerebello-cerebral circuit^48,52,64^. In line with this view, the differences found between case-cohort groups might as well reflect data that are consistent with the more contemporary network-based view of the neural correlates for cognitive dysfunction. Evidence in support of this view comes from large and high quality data-sets on a wide range of complex behaviors^65–67^. It is commonly suggested that there is a fundamental principle of complex systems that share macroscopic behavior (e.g. neurodevelopmental impairment after CbH), despite profound differences (e.g. group differences) in the microscopic details of the components of their systems or their interaction mechanisms^56^.

## Conclusion

Using advanced MRI and surface-based analysis, this study could not replicate reduced contralateral cortical volume in extremely preterm infants with CbH scanned at term as previously described within six CbH infants. Instead we found bilateral increase and decrease in cortical thickness and cortical surface area that varied across matched case-cohort groups. This study highlights that cerebello-cerebral development may be more complex than often previously assumed and is most likely modulated by multiple processes during the finely regulated brain maturation. Interestingly, this view has already been postulated in the mid-twentieth century by Verhaart and Wieringen-Rauws (1950) who concluded that despite prominent lesions the cerebellar-induced cortical atrophy “remains a very unsettling affair”. We propose that interferences about the developmental relationship between the cerebellum and the cortex should be drawn in respect to its complex interplay with timing and clinical circumstances, such as the size and location of the hemorrhage and potential clinical mediators and confounders.

## Data Availability

Inquiries about and requests for access to data generated and analyzed during this study should be directed to the corresponding author.
